# Care Partner Inclusion of People Hospitalized With Alzheimer Disease and Related Dementias: Protocol for a Mixed Methods Systems Engineering Approach to Designing a Health Care System Toolkit

**DOI:** 10.2196/45274

**Published:** 2023-05-16

**Authors:** Beth Fields, Catherine Still, Austin Medlin, Andrea Strayer, Alicia I Arbaje, Andrea Gilmore-Bykovskyi, Nicole Werner

**Affiliations:** 1 Department of Kinesiology University of Wisconsin-Madison Madison, WI United States; 2 Department of Design & Wellness Indiana University Bloomington, IN United States; 3 College of Nursing University of Iowa Iowa City, IA United States; 4 Department of Neurological Surgery University of Wisconsin-Madison Madison, WI United States; 5 Division of Geriatric Medicine and Gerontology, Department of Medicine Johns Hopkins School of Medicine Baltimore, MD United States; 6 BerbeeWalsh Department of Emergency Medicine University of Wisconsin-Madison Madison, WI United States

**Keywords:** caregiving, co-design, dementia, health care, systems, mixed methods

## Abstract

**Background:**

Research and policy demonstrate the value and need for the systematic inclusion of care partners in hospital care delivery of people living with Alzheimer disease and related dementias (ADRD). Support provided to care partners through information and training regarding caregiving responsibilities is important to facilitating their active inclusion and ultimately improving hospital outcomes of people living with ADRD. To promote care partners’ active inclusion, a toolkit that guides health systems in the identification, assessment, and training of care partners is needed. User-centered approaches can address this gap in practice by creating toolkits that are practical and responsive to the needs of care partners and their hospitalized family members and friends living with ADRD.

**Objective:**

This paper describes the study protocol for the development and refinement of the ADRD Systematic Hospital Inclusion Family Toolkit (A-SHIFT). A-SHIFT will provide health care systems with guidance on how to effectively identify, assess, and train care partners of hospitalized persons living with ADRD.

**Methods:**

The A-SHIFT study protocol will use a 3-aimed, convergent mixed method approach to iteratively develop and refine the toolkit. In Aim 1, we will use a systems-engineering approach to characterize patterns of care partner inclusion in hospital care for people living with ADRD. In Aim 2, we will partner with stakeholders to identify and prioritize health care system facilitators and barriers to the inclusion for care partners of hospitalized people living with ADRD. In Aim 3, we will work with stakeholders to co-design an adaptable toolkit to be used by health systems to facilitate the identification, assessment, and training of care partners of hospitalized people living with ADRD. Our convergent mixed method approach will facilitate triangulation across all 3 aims to increase the credibility and transferability of results. We anticipate this study to take 24 months between September 1, 2022, and August 31, 2024.

**Results:**

The A-SHIFT study protocol will yield (1) optimal points in the hospital workflow for care partner inclusion, (2) a prioritized list of potentially modifiable barriers and facilitators to including care partners in the hospitalization of people living with ADRD, and (3) a converged-upon, ready for feasibility testing of the toolkit to guide the inclusion of care partners of people living with ADRD in hospital care.

**Conclusions:**

We anticipate that the resultant A-SHIFT will provide health systems with a readiness checklist, implementation plan, and resources for identifying, assessing, and training care partners on how to fulfill their caregiving roles for people living with ADRD after hospital discharge. A-SHIFT has the potential to not only improve care partner preparedness but also help reduce health and service use outcomes for people living with ADRD after hospital discharge.

**International Registered Report Identifier (IRRID):**

DERR1-10.2196/45274

## Introduction

Persons living with Alzheimer disease and related dementias (ADRD) experience not only poorer health outcomes, but more hospital admissions and readmissions, longer hospital stays, and higher health care costs than older people who have not been diagnosed with ADRD [1-6]. Research and policy call for the systematic inclusion of care partners of people living with ADRD in hospitalizations to improve these health service use outcomes**.** Care partner inclusion has been associated with reduced readmissions and increased net savings [7,8]. Furthermore, when included in care, care partners report feeling more satisfied and less burdened [9]. Moreover, the policy has been mandated to better assist care partners in the hospital setting, including the Caregiver Advise, Record, and Enable Act. Across 44 states, hospitals are required to (1) provide patients with the opportunity to identify a care partner; (2) inform the patient and care partner when the discharge is to occur; and (3) provide the patient and care partner training regarding the discharge care plan [10]. Despite the supporting research and policy, care partners often report feeling unprepared to fulfill caregiving roles during and after a hospitalization of a family member or friend living with ADRD [11,12]. This is problematic as the 2022 National Strategy to Support Family Caregivers outlines federal, state, and local actions to help strengthen care partner services, including “recognizing them as essential partners in care teams of the person to whom they are providing support” (Outcome 2.1) [13].

Although research and policy work endorse the need for inclusive care practices, to our knowledge, no toolkit exists to guide health care systems when working with care partners of people living with ADRD [14]. To address this gap, in accordance with the National Institutes of Health Stage Model for Behavioral Intervention Development [15], we propose to conduct the critical Stage 0 and IA activities to develop the ADRD Systematic Hospital Inclusion Family Toolkit (A-SHIFT). We will use a user-centered approach to address each stage and to (1) uncover key hospital workflow intervention points, (2) identify and prioritize barriers and facilitators to care partner inclusion at multiple system levels (Stage 0), and (3) guide the transformation of these factors through stakeholder co-design into a comprehensive toolkit to help facilitate the inclusion of care partners of hospitalized people with ADRD, which is adaptable for use by different health systems (Stage IA). The following specific aims will be accomplished:

- Aim 1: describe the current practices around care partner identification, assessment, and training for hospitalized people living with ADRD.
- Aim 2: identify and prioritize health care system facilitators and barriers to the inclusion of care partners of hospitalized people living with ADRD.
- Aim 3: collaborate with stakeholders to co-design an adaptable toolkit to facilitate the inclusion of care partners of hospitalized people living with ADRD.

Toolkits, such as A-SHIFT can provide health care systems practical guidance regarding how to systematically include, assess, and train care partners of hospitalized people with ADRD. Further, A-SHIFT will be scalable to health care systems across the United States, providing the foundation for fulfilling the needs of care partners and alleviating their feelings of being unprepared to provide care following discharge.

## Methods

### Design and Conceptual Framework

**Figure 1:**
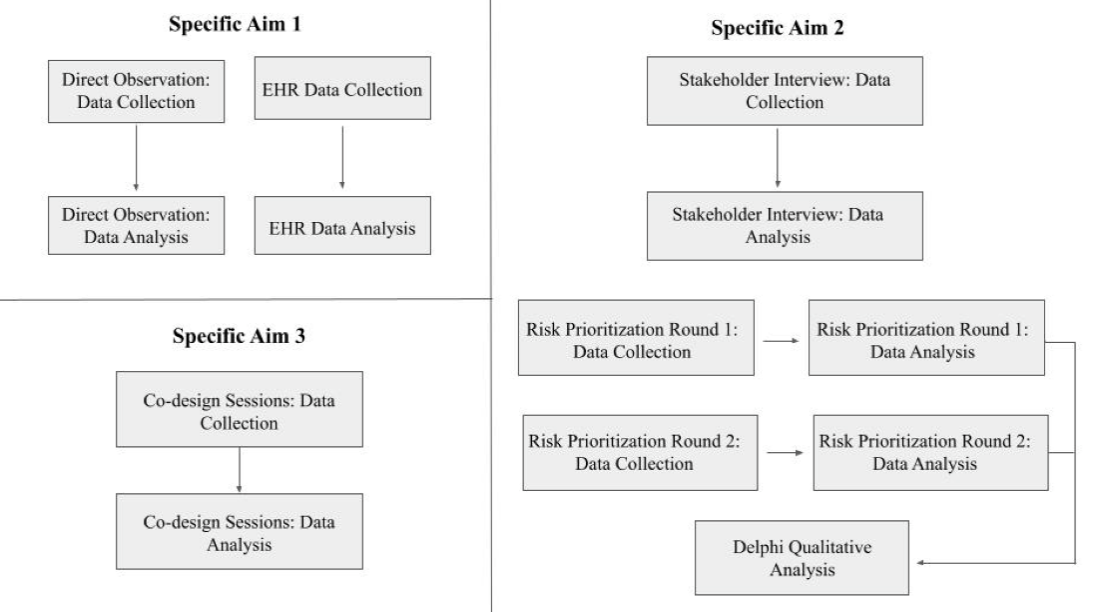
Overview of specific aims. EHR: electronic health record.

**Figure 2:**
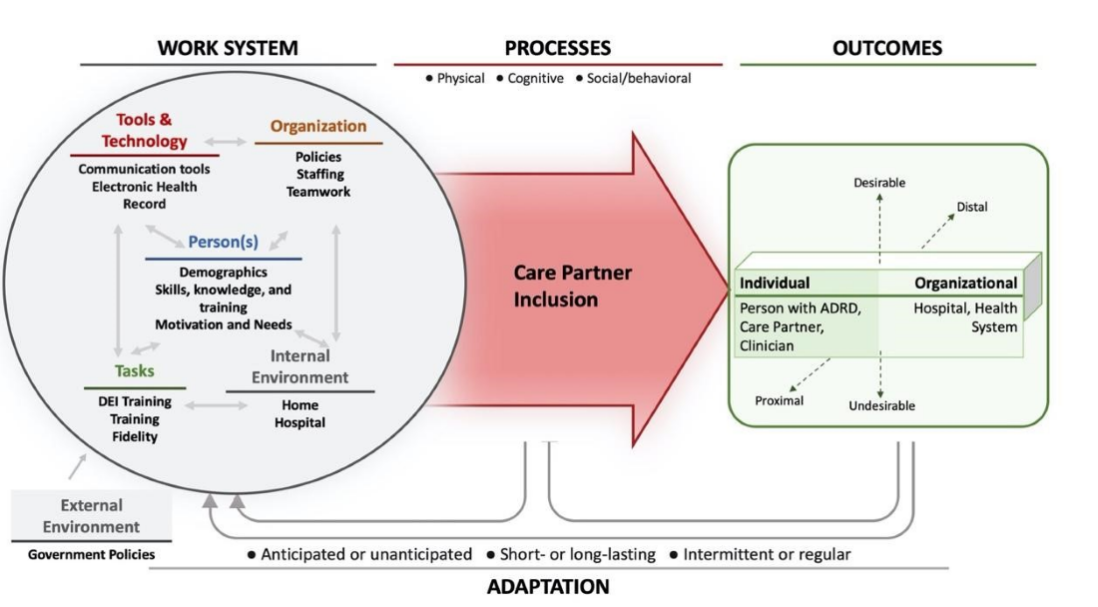
System Engineering Initiative for Patient Safety 2.0. ADRD: Alzheimer disease and related dementias; DEI: Diversity, Equity, and Inclusion.

Our interdisciplinary team will use a convergent mixed method approach [16] using systems engineering methods to conduct the necessary Stage 0 and IA activities of the National Institutes of Health Stage Model [15] to develop A-SHIFT across 3 aims (Figure 1). Our aims are guided by the System Engineering Initiative for Patient Safety (SEIPS) 2.0 model (Figure 2) [17]. SEIPS 2.0 has been used to design and implement health care processes in myriad health care settings [18-23]. SEIPS 2.0 expands upon Donabedian’s structure-process-outcome model [24] by providing a detailed and expanded structure (ie, work system) of interacting components (people [or teams]; tasks; tools and technology; organizational factors [eg, policies and teamwork]; physical environment [lighting, noise]; and external environment) that influence health care processes (eg, identify, assess, and train care partners of hospitalized people living with ADRD) and outcomes [4]. The model depicts a system that adapts to process changes and outcomes. SEIPS 2.0 will be used to guide data collection and analysis to ensure the identification of factors influencing care partner inclusion across all system components.

### Aim 1: Using Direct Observation and Electronic Health Record Data to Characterize Patterns of Care Partner Inclusion in Hospital Care for People Living With ADRD

#### Setting

We will collect observation and electronic health record (EHR) data from a large academic health care system in the Midwest. This system serves more than 600,000 patients each year with approximately 1750 physicians and 2100 staff at 7 hospitals and more than 80 outpatient sites. A major focus of this health care system is to put patients and their families, health plan members, employees, and community at the center of everything they do and create a model that ensures patient and family-centered care.

#### Direct Observation Sample

We will purposively select 4 diverse inpatient units from this health care system for direct observations, such as a trauma or medical surgical unit, an orthopedic unit, a family practice unit, and a neuroscience or stroke unit. These units allow for more heterogeneity of admitted patient conditions, providing us greatest exposure to various care partner needs.

#### EHR Sample

We will purposively select EHRs for patients across all hospitals in the health care system who meet our selection criteria: (1) aged >18 years with an ADRD diagnosis, (2) admitted to an inpatient unit, and (3) care partner identification, assessment, or training documented during patients’ hospital stay. To enhance rigor and reproducibility, there will be no exclusion based on sex, race, ethnicity, or HIV status. Records will be excluded for same-day surgery patients and patient discharge disposition that is not home (eg, skilled nursing facility).

#### Direct Observation Data Collection

We will capture care partner interactions on each selected unit using a structured SEIPS 2.0-based observation guide in Vosaic Software (Table 1) [25]. Observations will be conducted across 3 days in each unit. Each observation session will consist of a 3-hour block of time that represents morning, afternoon, and evening shifts to maximize understanding of different care points and care partners’ availability. In total, we will collect 27 hours of direct observations of direct care experiences (3 hours×3 days×3 units). Research team members will be trained to use the observation guide on department-owned laptops stored in a locked room. Training will consist of at least 2 meetings with the PI and research team members to discuss example observations for each code and review consent processes. Before starting data collection, the unit director will give a brief overview of the project to the clinicians on the unit and introduce the research team members. The team members will complete the observation guide for each interaction a clinician has with an admitted patient and any present care partners. Within a given interaction, team members will use Vosaic Software [25] to record each time they witness a code in the observation guide. To increase trustworthiness in our observations, each team member will create memos describing their observations of patient care and care partner inclusion, in addition to using the observation guide.

**Table 1:** Observation and electronic health record (EHR) data collection examples.

SEIPS^a^ component	Observation protocol	EHR fields
Work system organization internal environment	Are care partner policies, education, or trainings posted? What is the staff/patient ratio? Are there designated areas for care partners?	Hospital location (suburban, urban, and rural), size of unit
Work system personnel	Do providers invite care partners into patient interaction? How many providers did the care partner interact with?	International Classification of Diseases (ICD)-10 code, length of stay, discharge disposition, patient age, Patient sex, marital status, reason for admission
Process care delivery	At what point in the stay is the observation occurring: admission; during stay; discharge How do providers involve care partners: assessment; hands-on demonstration; verbal instruction; written instruction Were care partners notified of upcoming patient discharge?	Medical service (ie, provider type), patient or care partner education topic (eg, prescriptions, pain management, food and fluids, and preventing infections), patient or care partner taught (eg, patient, child, and spouse), patient or care partner education method (eg, oral instruction, demonstration, printed materials, and teach back)
Outcome patient	Do patients and care partners seem satisfied with their care experiences?	N/A^b^
Work system technologies	Do care partners have access to EHRs or other tools? Did providers document care partner interactions?	N/A

^a^SEIPS: System Engineering Initiative for Patient Safety.

^b^N/A: not applicable.

#### Direct Observation Data Analysis

Data will be summarized as frequencies and proportions, as appropriate. Frequencies will represent the number of times our study team observed a given phenomenon, such as the use of a care partner assessment by a provider. Proportions will provide a nuanced picture of the relative frequencies of certain codes, such as how often care partner education was initiated by the provider versus the care partner themselves. We will then create a SEIPS 2.0-based process map of the current hospital workflow to include care partners using both observation and memo data. We have developed methods for using SEIPS 2.0 to create process maps that simultaneously depict workflow and the system barriers associated with each stage of the workflow [26].

#### EHR Data Collection

We will select EHR fields that align with SEIPS 2.0 domains (Table 1). Data will be obtained from January 2019 to August 2022 to capture care practices before and during the COVID-19 pandemic. This time frame will allow us to better understand the impact of any visitor restrictions on care partner inclusion practices.

#### EHR Data Analysis

To calculate the sample size, we conducted an a priori power analysis using G*Power 3.1 [27]. For a chi-square goodness-of-fit test with a small effect size of 0.3, an α level of .05, a power level of 0.80, and 10 degrees of freedom (the maximum for any of our variables), a sample size of 181 was determined. We anticipate identifying several thousand patients in the hospital system who meet our inclusion criteria. We will not be able to gather EHR data from the same patients we observe due to the time it can take to record, process, and submit documentation for billing purposes. We will use SAS 9.4 [28] for data management and statistical analysis of the EHR data. We will conduct descriptive analyses of means, SDs, medians, and frequencies for all project variables.

#### Merging the Observation and EHR Data

We will use our direct observation data to identify ways in which to stratify the EHR care partner inclusion characteristics. For example, our direct observations may indicate that clinicians tend to include care partners in hospital care in distinct ways. We may also learn from our direct observations that patients with certain admitting conditions (ie, ICD-10 code) are more likely to have their care partners included in care. Depending on how we stratify care partner inclusion characteristics, we will use bivariate analyses: *t* test or ANOVA (continuous variables) and chi-square tests (categorical variables). We will also use classification and regression tree analysis to look for patterns in care partner inclusion across competing prediction variables. Classification and regression tree produces clear classification criteria and a visual “tree” showing the hierarchical relationships between variables within subgroups of the sample, which facilitates interpretation and direct translation to practice.

### Aim 2: Using Stakeholder Interviews and Risk Prioritization Methods to Identify and Prioritize Health Care System Facilitators and Barriers to the Inclusion of Care Partners of Hospitalized People Living With ADRD

#### Setting

Based on established relationships with UW Health, the University of Pittsburgh National Center on Family Support, and the Johns Hopkins University School of Medicine, we will recruit stakeholders from 3 geographical locations, Wisconsin, Pennsylvania, and Maryland. These locations will provide increased exposure to various health care system facilitators and barriers to the inclusion of care partners of hospitalized people living with ADRD.

#### Sample

Stakeholders will be purposefully selected from the following groups: (1) health care administrators and payers, (2) frontline clinicians (eg, hospital-based physicians, nurses, pharmacists, and therapists), and (3) hospitalized patients with ADRD and their care partners. These groups were selected because they will help our research team better understand dementia care in the hospital setting based on direct experiences. In each geographical location, we will recruit 5 health care administrators and payers, 5 frontline clinicians, and 5 patient and care partner dyads that meet eligibility criteria (Table 2).

**Table 2:** Sampling plan.

Stakeholder	Reason for inclusion	Eligibility criteria	Target, n
Health care administrators and payers	Knowledge and expertise related to the day-to-day operations of hospital, patient eligibility, enrollment, claims, and payment of health services	Have at least 5 years professional experience in position Speak and understand English	15
Frontline clinicians	Knowledge and expertise related to the delivery of health services	Have at least 5 years professional experience in their position Speak and understand English	15
Patients with ADRD^a^ and their care partners	Knowledge and experience related to receiving hospital care	Patient: Admitted to, or recently (within the last year) discharged from a hospital Diagnosed with some form of dementia Identified a care partner Care partner: provide unpaid care to a relative or partner with ADRD be at least 18 years of age or older speak and understand English	15 dyads

^a^ADRD: Alzheimer disease and related dementias

#### Stakeholder Interviews Data Collection

We will conduct semistructured interviews either face-to-face or over the telephone with stakeholders. The semistructured interview guide will be developed based on SEIPS 2.0 (Table 3) and will be tailored to each stakeholders’ experiences and responsibilities. Interviews will last up to 60 minutes and will be audio recorded, transcribed verbatim using department desktops in a locked lab space, and analyzed in NVivo 12 [29].

**Table 3:** Example System Engineering Initiative for Patient Safety (SEIPS) 2.0–guided interview questions.

SEIPS 2.0 domain	Stakeholder	Interview questions
Work system (Organization and Environment)	Health care administrators	In what ways, if any, does your organization demonstrate patient and family-centered care?
Process (Care Delivery)	Frontline clinicians	In what ways, if any, do you communicate discharge plans with care partners?
Outcome (Patient)	Patient and care partner dyad	In what ways do you feel you were prepared to carry out health care tasks once home?
Work system (Technologies)	Frontline clinicians	In what ways, if any, are care partner inclusion practices documented within the EHR^a^?
Outcomes (Organizational)	Wisconsin-based payers	In what ways, if any, are care partner inclusion practices evaluated?

^a^EHR: electronic health record.

#### Stakeholder Interviews Data Analysis

We will perform directed content analysis [30] guided by SEIPS 2.0. The research team will review a subset (ie, 2-3) of interview transcripts and use SEIPS 2.0 to develop high-level codes of facilitators and barriers to the systematic inclusion of care partners in hospital care. For example, under the high-level code of “tools and technology,” there will be a subcode for barriers and for facilitators. We will use these codes as guides to review subsequent transcripts and revise codes as needed until the codebook is formalized. Two team members will apply the codebook to independently code 20% of the data. We will then use the coding comparison query function in NVivo 12 to measure intercoder reliability. A threshold will be set at 80% agreement based on suggested values of intercoder reliability [16]. The team members will discuss disagreements and continue to code 2-3 interviews independently until this threshold is reached. Once the threshold is met, team members will finish the remaining coding. After the qualitative coding of interviews is completed, frequencies of code usage and percentages of all coded texts will be computed to quantify the degree to which certain aspects of SEIPS 2.0 were represented.

#### Risk Prioritization Data Collection

We will use a modified Delphi approach [31] where the same stakeholders who participated in interviews will conduct risk prioritization ratings in 2 rounds. We will conduct the first round of the Delphi over a 1-month period. Each group of stakeholders will be sent a web-based questionnaire using the Qualtrics survey platform. They will be asked to rate each barrier and facilitator identified from the stakeholder interviews on the following three categories, each on a 5-point scale: (1) frequency of occurrence (very high frequency to very low frequency), (2) level of importance in influencing the inclusion of care partners in hospitalizations (very high importance to very low importance), and (3) likelihood that the factor is modifiable (very high to very low). We will also provide an optional free-text field for each entry so that stakeholders can provide comments about each barrier and facilitator or their rating decision. Stakeholders will also be able to enter suggestions for additional content that they think is missing and important to add. The free-text data will allow for an in-depth understanding of the rationale underlying ratings and will also serve as a member-checking process to ensure validity of the data. Following the same procedures from round 1, we will conduct round 2 of the risk prioritization. In this round, stakeholders will be provided with a reminder of what their ratings were in round 1, and will also be provided with, and asked to consider, the combined responses from all other stakeholders in round 1.

#### Risk Prioritization Data Analysis

We will use descriptive statistics to summarize responses. We will then assign each potential item an overall prioritization score by multiplying the individual scores from each category. Factors that received over 75% consensus among stakeholders will be considered to have met consensus in round 1. Items will be considered to have met consensus when at least 75% (34/45) of stakeholders agree within 1 point on the Likert scale, for example, over 75% of participants rate an item as likely (4) or extremely likely (5). Factors that receive less than 75% consensus will be used in the second round of ratings. A systematic review of Delphi studies indicated that 75% was the median threshold for percent agreement-based consensus across studies [32]. Factors that do not reach over 75% consensus after round 2 will not be included in the prioritization index. However, factors not reaching consensus will be presented for consensus discussion by design teams in Aim 3.

The free-text data will be uploaded to NVivo 12 for qualitative analysis. We will use an interdisciplinary team-based thematic analysis [33] to identify themes related to stakeholders’ rationale for ratings. Coding will be performed by research team members independently, and themes will be developed iteratively following the 6 phases outlined by Clarke and Braun [33]. In phase 1, we will familiarize ourselves with the data from both rounds of risk prioritization. In phase 2, we will thoroughly code the data using a systematic process guided by SEIPS 2.0. Team discussions will be used to reach a consensus on codes throughout this phase [34]. We will then move into phase 3, which involves a generation of initial, or candidate, themes and an exploration of relationships between themes. This phase will produce a thematic map to display these relationships. In phase 4, we will assess our candidate themes against the coded data to evaluate how well the themes fit the coded data. If a good fit is determined, the research team will assess candidate themes against the entire data set, editing the themes as needed. This process will continue until the research team members have come to a consensus and are satisfied with the definitive themes. Phase 5 will involve generating definitions for each theme. This process will conclude with phase 6, which consists of writing up the findings of this analysis [33].

### Aim 3: Using a Co-design Process to Iteratively Develop an Adaptable Toolkit to be Used by Various Health Care Systems to Facilitate the Inclusion of Care Partners of Hospitalized People Living With ADRD

#### Setting and Sample

We will convene 2 co-design groups using the same recruitment settings and stakeholders from Aim 2. One group will consist of patient or care partner dyads and the other group of health care administrators, payers, and clinicians. Each design team will have approximately 5 to 7 stakeholders, which is within the standard range of stakeholders used in participatory design research [35]. The odd number of stakeholders allows for a tie-breaking perspective in the groups. The small number of stakeholders in groups (ie, rather than 10-14 in one group) reduces the potential for groupthink (ie, by having products from 1 group evaluated by the other) and ensures that all stakeholders have ample time to provide their perspectives [36].

#### Co-design Data Collection

Design teams will complete 5 co-design videoconference sessions occurring in parallel across 4 months, with 3 weeks between each session. The design process can be conceptualized as a funnel [6] in which we start with myriad divergent ideas and then begin to work toward convergence through the sessions. In session 1, we will present the teams with the prioritized list from our risk prioritization process completed in Aim 2. We will lead discussion, interpretation, and respectful debate among design team members to begin to generate health care system process changes to optimize the inclusion for care partners of hospitalized people living with ADRD. We will ensure progress toward solution convergence from session to session. The design teams will conduct sessions independently, but in between sessions will swap solutions and provide feedback, which we will summarize and present in subsequent sessions. Each session will last up to 90 minutes. Sessions will be audio recorded for subsequent analysis. Sessions will be held virtually to support retention using our established web-based process for participatory design, which has been a successful alternative to in-person design sessions.

#### Co-design Data Analysis

Analysis of design session outputs such as sketches, use of case scenarios, ratings, research team memos, and stakeholder notes will occur both within and between sessions. Within sessions, we will guide stakeholders in generating, grouping, and converging upon design solutions. Within the 3-week period between sessions, we will review the audio recording of the session and use the rapid identification of themes from audio-recordings method [37] to identify design specifications. We will combine the themes from the rapid identification of themes from audio recordings with synthesized outputs from the session (eg, sketches and ratings) to provide inputs to stakeholders at the following design session. Following the design session completion, we will continue to refine the A-SHIFT, applying input from the final design session, and performing member checking by reviewing changes with the stakeholder groups [38].

### Ethics Approval

The study was approved by the Institutional Review Board (IRB) at the University of Wisconsin-Madison on March 23, 2023 (approval no. 2022-0024). All protocol modifications will be reviewed and approved by the IRB as needed. We will obtain a waiver of signed consent using a process that employs an IRB-approved information sheet.

## Results

We anticipate this study to take 24 months to complete. All 3 aims will take place between September 1, 2022, and August 31, 2024. We expect the following results:

- Aim 1 expected results: observations and EHRs will provide 2 views of care partner interactions—whether care partners are included in hospital care and how inclusion is documented. Analyses are in progress and will result in the identification of optimal points in the hospital workflow for care partner inclusion that will be presented during stakeholder interviews in Aim 2.
- Aim 2 expected results: a prioritized list of potentially modifiable barriers and facilitators to including care partners in hospitalization of people living with ADRD will be presented to the Aim 3 co-design teams. This information will provide the basis for designing A-SHIFT.
- Aim 3 expected results: results will provide a converged-upon, ready for feasibility testing of A-SHIFT to guide inclusion of care partners in hospital care of people living with ADRD. We anticipate that the A-SHIFT will include strategies on when and how to identify care partners in hospital care, tools for assessing the needs of care partners and their caregiving preparedness, methods for how to train care partners to fulfill caregiving roles for people living with ADRD after hospital discharge, a health care system readiness checklist and implementation plan, and suggested measures of successful implementation.

Study results will be disseminated in peer-reviewed journals and presented at annual scientific meetings. We will also share the results of this study directly with stakeholders by developing infographics. These will be distributed through dementia caregiving networks and established partnerships.

## Discussion

Despite research and policy calling for the systematic inclusion of care partners in hospitalizations to improve outcomes of persons living with dementia, no toolkit exists to guide health care systems on how to effectively identify, assess, and train care partners.

We will be using a user-centered, systems engineering approach to develop A-SHIFT that is well-suited to address real-life barriers to care partner inclusion in health systems. Our systems-engineering approach to development increases the likelihood of the successful implementation of A-SHIFT in health systems. In using SEIPS 2.0, we will acquire a comprehensive understanding of the work systems, processes, and outcomes related to care partner inclusion that will enable us to create a toolkit that addresses barriers in each of these areas. SEIPS is an inherently user-centered model, which places the people at the center, and as such, promotes the design of systems that optimally support the people in the system. Additionally, we will use a mixed methodology in our data collection, which will provide us with a rich understanding of current practices of care partner inclusion that quantitative and qualitative methods could not provide on their own. Most importantly, our methodology involves stakeholders from multiple disciplines, sites, and geographical areas, which will increase the likelihood of A-SHIFT being implemented across a range of health systems. Our user-centered approach, including engaging stakeholders as co-designers, has been recognized as an important approach by the World Health Organization [39], the National Institute of Standards and Technology [40-42], and the International Standards Organization [43,44]. The user-centered approach is the epitome of person-centered methodologies [45] and is critical to the design of an A-SHIFT that meets the needs of all stakeholders.

We may find that stakeholders will not wish to participate in this study, or that we cannot retain them as expected. We have infrastructure, however, for successfully recruiting and retaining stakeholders, and strong partnerships to expand recruitment strategies. For example, our team has secured funding to provide stakeholders compensation and we have access to technology support. We have also designed our approach to reduce stakeholder burden. In particular, we will use phone and videoconferencing to conduct interviews and design meetings and we will mail stakeholders remuneration. Although we have adapted our risk prioritization methods to be delivered using a Delphi approach as a COVID-19 precaution, we recognize that this approach is slower than traditional in-person focus groups or meetings. Our research team has also fostered collaborative partnerships with representatives from 3 different health care systems to increase the generalizability of A-SHIFT. Although the direct observations and EHR data from Aim 1 will only represent 1 academic medical setting, these results will serve as a basis for discussion with stakeholders from all 3 systems in Aims 2 and 3.

A-SHIFT will provide diverse health care systems guidance on how to identify, assess, and train care partners of hospitalized people living with ADRD. Our inclusion of stakeholders from health systems in different geographical locations will promote the creation of a toolkit that is more generalizable across health systems. The development and refinement of A-SHIFT are critical for the next planned R-series grant proposals, which will address Stage 1B feasibility testing followed by hybrid efficacy or effectiveness testing of A-SHIFT’s effectiveness at improving care partner preparedness and reducing avoidable hospital readmissions by people living with ADRD. Care partners that are well-prepared have the potential to increase their own well-being and also help optimize health and service use outcomes for people with living ADRD after hospital discharge. Furthermore, we hope our study protocol can be used as a model for researchers to develop additional toolkits to target key areas of concern across health care systems. Our comprehensive, systems-engineering approach promotes the creation of user-centered toolkits that meet all stakeholder needs to foster sustainable change in health care system processes to ultimately improve patient and care partner outcomes.

## Abbreviations

ADRD: Alzheimer disease and related dementias
A-SHIFT: ADRD Systematic Hospital Inclusion Family Toolkit
EHR: electronic health record
IRB: institutional review board
SEIPS: System Engineering Initiative for Patient Safety

## Data Availability

The data sets generated and analyzed during this study will be available from the corresponding author on reasonable request.
